# American Academy of Dental Science

**Published:** 1879-02

**Authors:** Edward N. Harris

**Affiliations:** No 5 Park St., Boston


### Proceedings of Societies.

### AMERICAN ACADEMY OF DENTAL SCIENCE.

The eleventh annual meeting of the American Academy of Dental Science was held in Boston on Wednesday, October 30th, at the Hotel Brunswick,

There was a very good attendance, including many eminent members of the profession.

The meeting was called to order at half-past ten o'cloek, by the President, Dr. E. G. Tucker, who delivered a brief address of welcome, after which came the regular order of business.

Drs. G. L. Parmelee, of Hartford, Conn., J. Adams Bishop, of New York, and L. Tracy Sheffield, of Paris, were elected members. Dr. Joseph E. Fisk, of Salem, Mass., was elected an honorary member.

The following gentlemen have been elected to membership during the year: Drs. G. H. Ames, and F. G. Eddy, of Providence, R. I., F. H. Rehwinkle, of Chillicothe, Ohio, and C. A. Marvin, of Brooklyn, N. Y.

Dr. Elbridge Bacon, of Portland, Main, was elected to honorary membership.

Officers elected for the ensuing year:

President, Dr. E. G. Tucker.

Vice-President, Dr. J. L. Williams.

Recording Secretary, Dr. E. P. Bradbury.

Corresponding Secretary, Dr. E. N. Harris.

Treasurer, Dr. L. D. Shepard.

Librarian, Dr. J. T. Codman.

Board of Censors; Drs. T. N. Chandler, W. W. Codman and D. M. Parker.

A communication was received from the Massachusetts Dental Society inviting the Academy to meet with the other dental societies of New England, in joint convention, to be held in the city of Boston in June, 1879. The invitation was accepted.

The following resolutions were presented and after discussion were adopted:

*Resolved*, That the use of public prints for advertising pretended professional merit, is derogatory to the dignity of the profession, and should be strongly discountenanced by its members.

*Resolved*, That among regular practitioners any disparagement of their fellows to patients or the public, is a serious infraction of true ethics, tends to lower the profession in public estimation and to debase the individual detractor, and should be condemned by all who have the best interests of the profession at heart.

The Academy having completed the routine business, took a recess until 2 p. m.

At 2:30 p. m., Charles W. Elliot, LL. D., president of Harvard University, delivered the annual address before a large number of the profession, including many who were present by invitation of the Academy.

The following is but a brief synopsis of the address, which will soon be published in full. It was of an informal nature, delivered in a graceful and eloquent manner and without notes.

President Elliot took for his theme, "The defects apparent in the American system of Dental Education." He said that the development of the dental profession in the seventy years of its existence in the United States had been remarkable, but as the development of a profession requires centuries, dentistry could not be expected to possess all the safeguards from injurious influences which have been attained by older professions.

The progress of dental science in this country not only has been extraordinarily rapid, but on the whole satisfactory, and the causes of this, the orator said, were the American inventive genius, the hospitality of the American mind to novelties, and the soft, unphosphatic diet to which Americans are fond. Besides this, every cultured American visited a dentist twice a year, while an Englishman only went when his tooth pained him; the former sought relief for future time, the latter for the present.

Mr. Elliot then took up the question, Is there real ground for the anxiety which exists for the improvement of the status of the profession, and especially dental education?

From the great increase in the number of dentists, and of patients, the increase in periodical literature and standard works, and the increase in the organized means of education, he thought there was real ground for that anxiety which manifests itself at the present day in dental literature and discussions.

The improvement must be brought about in a great measure through the organized means for the education of the profession. Our dental schools demand no preliminary examination, while those of England require several university examinations, and this simple fact will in time determine the superiority of the profession in England.

He argued that besides the preliminary examination of candidates for admission, that the course of dental study should be three instead of two years long, and said that a change in this direction is essential to the establishment of the dental profession on an equality with other professions, There should be no admission of the substitute of five years of practice for any portion of the period of study. There should be a certificate of attendance upon what might be called "private instruction," deposited in advance, and the practitioner granting such certificate should be required to show that he has proper facilities for instruction.

The exclusion of ignorant men from the profession was next discussed, the orator giving the proposition that to secure this by means of a public registry, as in other countries, is not practical in America; the people, or their representatives in legislative halls, are not competent to decide questions of science or of the higher education. Educated public opinion and not legislation would in time reduce the evil,

He commented unfavorably on the recent new departure of several reputable medical schools, which advertise to confer both the medical and dental degree for three years of study; and argued that as not more than three-fifths of the studies of these two courses were in common, and that as a three years' course was absolutely necessary to the medical degree, the two degrees could not be given in that period without lowering the standard of both. While he hardly expected at present that the large proportion of dental students would be educated in the thorough way of first obtaining the medical and then the dental diploma, he hoped that the efforts of the profession would be directed to the improvement and upraising of the dental schools.

He urged the profession to emulate the zeal of the medical profession in its research and scientific study, and also in their noble example of gratuitous practice. He advised the necessity of strict professional etiquette in dealing with patients of other practitioners, and spoke of the value of associated action of the profession in the common pursuit of common knowledge, and of the value of recorded experience and observation as a help to those coming after, and above all he placed the three aids, research, teaching and gratuitous labor as establishing and enobling the profession.

The address was received with close attention, and at its close a vote of thanks to President Elliot, coupled with a request for permission to publish, was unanimously passed.

An essay by Dr. Henry S. Chase, of St. Louis, on "Filled Teeth as Galvanic Batteries," was read by Dr. G. T. Moffat, a letter having been received by him from Dr. Chase stating that he was unable to be present. Dr. Chase, after announcing his belief in the theory of Dr. S. B. Palmer, of New York, that every tooth having a metallic filling was a galvanic battery, procecded to recount several experiments made by himself which had convinced him of the truth of this theory. The conclusions arrived at were that dentos was positive to gold, gold and platinum, amalgam, tin and lead. Each of these metals united to dentos formed a battery, the power of which could be stated thus:

| | |
|---|---|
| With gold and gold and platinum, | 100 |
| Amalgam, | 66 |
| Tin, | 50 |
| Lead, | 30 |

With Hill's stopping there is no action, and with oxychloride of zinc the tooth becomes negative and the filling positive. In concluding he said that the perfect filling was yet to be discovered. It must not be negative to dentos, it must not be positive to dentos, and it must be plastic.

The essay elicited quite a lively discussion, after which the Academy adjourned, and the members with the invited guests partook of the eleventh anniversary dinner at five o'clock, p. m., at the Hotel Brunswick.

EDWARD N. HARRIS,
Corresponding Secretary,
No 5 Park St., Boston.

